# Prevalence and correlates of low back pain among undergraduate medical students in Serbia, a cross-sectional study

**DOI:** 10.7717/peerj.11055

**Published:** 2021-03-08

**Authors:** Irena Ilic, Vesna Milicic, Sandra Grujicic, Ivana Zivanovic Macuzic, Sanja Kocic, Milena D. Ilic

**Affiliations:** 1 Faculty of Medicine, University of Belgrade, Belgrade, Serbia; 2 Department of Dermatovenerology, Faculty of Medical Sciences, University of Kragujevac, Kragujevac, Serbia; 3 Institute of Epidemiology, Faculty of Medicine, University of Belgrade, Belgrade, Serbia; 4 Department of Anatomy, Faculty of Medical Sciences, University of Kragujevac, Kragujevac, Serbia; 5 Department of Social Medicine, Faculty of Medical Sciences, University of Kragujevac, Kragujevac, Serbia; 6 Department of Epidemiology, Faculty of Medical Sciences, University of Kragujevac, Kragujevac, Serbia

**Keywords:** Low back pain, Medical students, Prevalence, Risk factors

## Abstract

**Background:**

Low back pain (LBP) is a serious health problem among medical students. We aimed to investigate the prevalence and associated factors for LBP among Serbian medical students.

**Methods:**

A cross-sectional study was performed among medical students at one University in Serbia. Data was collected by a self-reported questionnaire. Logistic regression was used to determine the factors associated with LBP; results from the analyses were expressed as odds ratios (OR) with 95% confidence intervals (CI).

**Results:**

The study sample comprised 499 medical students, giving a response rate of 92.9%. The mean age of the participants was 22.0 ± 2.2 years (range 18–34). Overall, 20.8% (104/499) of medical students had LBP at the time of study. Cigarette smoking (OR = 2.5, 95% CI [1.5–4.2], *p* = 0.001), stress during classes (OR = 1.8, 95% CI [1.1–3.0], *p* = 0.039), incorrect sleeping position (OR = 1.9, 95% CI [1.2–3.1], *p* = 0.006) and family history of LBP (OR = 1.6, 95% CI [1.1–2.7], *p* = 0.047) were independently associated with high prevalence of LBP at the time of the study.

**Conclusions:**

LBP is a highly prevalent health problem in medical students in Serbia. The association between LBP and cigarette smoking, stress during classes, incorrect sleeping position and LBP in family history has been observed in medical students.

## Introduction

Low back pain (LBP) is one of the most common health problems and it affects people of all ages, from children to elderly (Al Amer, 2020; Chiwaridzo, Chamarime & Dambi, 2018; Hoy et al., 2012; Duthey, 2013). According to the definition accepted by the World Health Organization, pain is defined as an unpleasant sensory and emotional experience associated with, or resembling that associated with, actual or potential tissue damage (Raja et al., 2020). Due to its negative effects on physical and mental health, LBP is a major socio-economic problem for the community (Alonso-García & Sarría-Santamera, 2020; Wu et al., 2020).

Many studies have indicated that LBP is highly prevalent among healthcare workers (Al Amer, 2020), with some suggesting that in some of them LBP began before they started working (Karahan et al., 2009). However, very few studies evaluated back pain among medical students. Additionally, the available literature suggests that there is a high prevalence of LBP among students of medicine (Tavares et al., 2019; Chiwaridzo, Chamarime & Dambi, 2018; Noormohammadpour et al., 2019; Algarni et al., 2017; Vincent-Onabajo et al., 2016; Kamada et al., 2016). Circumstances which might be responsible for making this population prone to the occurrence of LBP are related to the highly demanding medical school curriculum (Algarni et al., 2017; Dighriri et al., 2019). Consequently, medical students are exposed to a sedentary lifestyle devoid of physical activities, as well as to stress and long periods of standing during clinical training. Further on, presence of LBP in medical students can affect their daily life, class attendance, academic success and future career (Amelot et al., 2019). Literature suggests that medical students in higher semesters, who have more practical activities in hospitals, have a higher LBP prevalence (Algarni et al., 2017; Vincent-Onabajo et al., 2016; Kamada et al., 2016; Alshagga et al., 2013; Aggarwal et al., 2013; Chibnall, Tait & Jovel, 2014; Hafner, Milek & Fikfak, 2018).

Some studies have suggested a link between LBP and some risk factors, including age, education, obesity, smoking, hereditary factors, prolonged standing, injury, stress, anxiety, depression, poor interpersonal relationships and lack of social support (Dighriri et al., 2019; Dionne et al., 2018; Hafner, Milek & Fikfak, 2018; Mehrdad et al., 2016). However, results of some others studies regarding the association between demographic characteristics and risk of LBP among medical students have been very inconsistent (Tavares et al., 2019; Al Amer, 2020; Algarni et al., 2017).

According to the available literature, only one study in this field was carried out in Serbia (Vujcic et al., 2018), where students of only one year of study were included in the research. Improving knowledge about the prevalence of LBP and factors associated with LBP in our medical students is important since it allows the assessment of the impact of this disease in this population group and can also help organize measures necessary for prevention and control. The objectives of this study were to determine the prevalence of LBP among medical students at one Serbian University and to investigate the potential factors associated with LBP prevalence.

## Materials and Methods

### Study setting

This study was carried out at the Faculty of Medical Sciences, University of Kragujevac, a state university in central Serbia. The study programme at the integrated academic studies for doctor of medicine is organized in semesters; there are 12 semesters in total during the 6 years of studies. The teaching process is realized through lectures, laboratory sessions, other forms of lecturing and six-year long professional practice. The teaching process is held by the use of interactive programmes oriented toward the students, providing conditions for a greater engagement of the students during lectures, with constant checking of their knowledge. The curriculum provides the means of knowledge evaluation according to the law on higher education: at least 30% of a grade is accomplished through the pre-final examination activities. The contents of the courses of the study programme are based on the latest knowledge in adequate disciplines, and oriented towards patients as the center of interest of theoretical and practical medicine. The methods of teaching are provided problem-oriented lectures and small-group work. A relatively small number of medical students (a total of 536 freshmen, from 2013 to 2018) are enrolled in the programme at the integrated academic studies of medicine, with the purpose of succeeding in fulfilling the set standards of quality. All teaching modalities (lectures, seminars, exercises) are mandatory, which encompasses regular students’ names call-outs to determine attendance to all academic activities. The research was conducted during November–December, 2018. The questionnaire was filled out by each participant during their classes at the medical faculty. All classes were visited by the researchers during the data collection period, in order to inform students about the goal of research and provide assistance during the filling out of the questionnaire, which included providing clarifications to any questions which participants might have had about the items in the questionnaire.

### Study design

The research was carried out using a cross-sectional design using a self-administered questionnaire. Only those respondents who gave voluntary, informed written consent for participation in the study were included.

### Study population

The research involved students of medicine at only one of the faculties of medicine in Serbia. All students who met the inclusion criteria were eligible to participate in the study.

All full-time medical students were considered for inclusion in the study. Eligible medical students were aged over 18 years, were fluent in spoken and written Serbian language. Also, the inclusion criteria included voluntary agreement to participate in the study. Reasons for ineligibility were age <18 at the time of recruitment, not speaking or writing in Serbian language, any other reason that did not allow or hindered participation in the study or refusal to participate in the study.

Responses to the questionnaire were received from 508 medical students (response rate was 92.9%). Responses which were not valid or fully complete (total of nine questionnaires) were not analyzed due to a lack of data. The reason for non-response was well documented in the study, and this was mostly lack of interest (notified by returning a form included in the letter with the initial study invitation). Finally, the participation rate was 91.2% (499/547). The nonparticipant group comprised 39 medical students who did not attend the classes at the time of the study, and about whom there is no data due to the anonymity of participants.

### Data collection

A self-reported, anonymous questionnaire was distributed to each participant. All study participants provided written informed voluntary consent prior to taking part in the study. After accepting to participate in the study, medical students completed the questionnaire, which took approximately 20 min.

For students, LBP was defined as ‘pain in the area on the posterior aspect of the body from the lower margin of the twelfth ribs to the lower glutaeal folds, that lasts for at least one day, with or without pain referred into one or both lower limbs’ (Duthey, 2013; Hoy et al., 2012). The definition of LBP was given verbally to medical students. In our survey, the case of LBP was defined as a positive response to the appropriate question, which was as follows: Do you NOW have lower back pain? No/Yes.

Questionnaire collected basic information such as age, gender, the study year, total overall grade (from 6 to 10), level of study, completed secondary school (Statistical Office of the Republic of Serbia, 2020). Further on, the questionnaire contained questions on the exposure to factors potentially associated with LBP, such as the habit of cigarette smoking, history of motor vehicle accidents, studying conditions (injuries at faculty, prolonged standing at faculty, prolonged sitting at faculty, incorrect body posture, lifting/moving heavy loads, stress during classes, conflicts at faculty), circumstances outside the faculty such as recreational sports, incorrect sleeping position, housework (window washing, etc.), house chores (ironing, etc.), other domestic tasks, weather (e.g. cold, humidity, …), draft, LBP in family history, other factors. The body mass index (BMI) was calculated based on self-reported weight and height. Degree of obesity was estimated as follows: BMI less than 18.5kg/m2 denoted underweight, 18.5–24.9 meant normal or healthy weight, 25.0–29.9 denoted overweight, while subjects with BMI 30.0 and above were considered obese (World Health Organization, 2000). As ‘other domestic tasks,’ students most often reported vacuum cleaning and dusting, hanging curtains and dishwashing. As ‘other factors,’ students most often reported computer usage, studying, watching television. Data concerning personal medical history included information on depression, anxiety, some other psychiatric illness, rheumatic disease, arterial hypertension, ischemic heart disease, diabetes mellitus, thyroid disease, asthma, anemia, migraine, sinusitis, myopia. Personal medical history was estimated as positive in the presence of either one or multiple diseases (such as depression, anxiety, chronic rheumatic diseases, hypertension, diabetes mellitus, thyroid disease, surgical interventions, etc.). Comorbidity represented the presence of more than one disease in personal medical history.

### Ethical considerations

All procedures performed in studies involving human participants were in accordance with the ethical standards of the institutional and/or national research committee (the Ethics Committee of the Faculty of Medical Sciences, University of Kragujevac, Ref. No.: 01-13459) and with the 2013 Helsinki declaration and its later amendments or comparable ethical standards. Informed consent was obtained from all individual participants included in the study.

### Data analyses

A descriptive analysis was performed for categorical variables by using absolute and relative frequencies. The association between factors potentially associated with LBP and LBP in medical students was investigated using univariate logistic regression. Multivariate logistic regression was used to determine the independent factors associated with LBP. Results from the logistic regression analyses were expressed as odds ratios (OR) with 95% confidence intervals (CI). Multivariate logistic regression models were made for all variables that were related to LBP in univariate analyses at a *p* value of <0.10. This analysis used the `enter` method (default with the menu system). Models fit were assessed by the Hosmer-Lemeshow test of goodness of fit and Cox and Snell’s and Nagelkerke’s Pseudo R square measures. The whole model was statistically significant (Omnibus goodness of fit test: Chi-square = 47.09, df = 8, *p* <0.001; Hosmer-Lemeshow goodness of fit test: Chi-square = 5.70, df = 8, *p* = 0.681). Cox and Snell’s and Nagelkerke’s Pseudo R square measures were 0.09 and 0.14, respectively. Among the observed variables, multicollinearity was examined with Variance Inflation Factors (VIF). Our results show that all independent variables had a VIF near 1, indicating that there is no correlation between this independent variable and any others. A test for linear trend in risk was based on the logistic regression model.

Statistical significance was accepted at the level of *p* < 0.05. All statistical analyses were conducted with SPSS 20.0 (SPSS, Chicago, IL, USA).

## Results

### Participant characteristics and prevalence of LBP

Based on the previously described inclusion and exclusion criteria, 508 medical students were recruited into the study group. Of that number, nine questionnaires were excluded from the data analysis due to invalid or incomplete answers. Finally, a total of 499 eligible participants were included in the study. The participation rate was 91.2% (499/547). Four hundred ninety nine medical students were evaluated: of the participants, 161 (32.3%) were male and 338 (67.7%) were female, while the mean age was 22.0±2.2 (range 18–34) years (Table 1). The proportion of students at clinical level of study (from 7th to 12th semester) was higher than the proportion of students on preclinical level - those in the first 6 semesters (54.5% vs. 45.5%). More than half of medical students (64.3%) previously completed secondary medical school. The proportion of students with overall grade ≥8 is higher (69.0%) than the proportion of students with lower overall grade (15.8%), with note that freshmen have not been graded yet. Overall, 20.8% (95% CI [17.6–24.0]) of medical students reported suffering from LBP at the time of answering the questionnaire.

**Table 1:** Basic characteristics of study participants.

Characteristics	Number (*N* = 499)	(%)
Age (years)		
<22	297	59.5
≥22	202	40.5
Mean ± SD (Range)	22.0 ± 2.2 (18–34)	
Gender		
Male	161	32.3
Female	338	67.7
Completed secondary school		
Grammar school	178	35.7
Secondary medical school	321	64.3
Study year		
1st	76	15.2
2nd	74	15.0
3rd	77	15.4
4th	92	18.4
5th	84	16.8
6th	96	19.2
Total overall grade		
None (freshmen)	76	15.2
<8	79	15.8
≥8	344	69.0
Level of study		
Preclinical	227	45.5
Clinical	272	54.5
Low back pain		
No	395	79.2
Yes	104	20.8

### Factors associated with prevalence of LBP

Tables 2 and 3 demonstrate the results of the univariate analyses concerning the factors associated with prevalence of LBP. No significant differences were observed in the LBP prevalence in medical students by age, gender, completed secondary school, study year, cumulative overall average grade, level of study (Table 2).

**Table 2:** Low back pain in medical students: univariate regression analysis.

Variable	Number (*n* = 104) (%)	OR (95% CI)	*P*
Age (years)			
<22	65 (21.9)	1.0*	
≥22	39 (19.3)	0.9 [0.5–1.3]	0.487
Gender			
Male	27 (16.8)	1.0*	
Female	77 (22.8)	1.46 [0.9–2.4]	0.124
Completed secondary school			
Grammar school	38 (21.3)	1.0*	
Secondary medical school	66 (20.6)	0.9 [0.6–1.5]	0.954
Study year			
1st	17 (22.4)	1.0*	
2nd	18 (24.3)	1.1 [0.5–2.4]	0.777
3rd	13 (16.9)	0.7 [0.3–1.6]	0.394
4th	23 (25.0)	1.2 [0.6–2.4]	0.690
5th	19 (22.6)	1.0 [0.5–2.1]	0.970
6th	14 (14.6)	0.6 [0.3–1.3]	0.190
*P* trend			0.452
Total overall grade			
None (freshmen)	17 (22.4)	1.0*	
<8	12 (15.2)	0.6 [0.3–1.4]	0.254
≥8	75 (21.8)	0.9 [0.5–1.7]	0.914
*P* trend			0.405
Level of study			
Preclinical	48 (21.1)	1.0*	
Clinical	56 (20.6)	0.97 [0.63–1.49]	0.879

**Notes:**

* Reference category.

Abbreviations: OR, Odds Ratio; CI, Confidence Interval; *P*, Probability value (according to logistic regression); *P* trend, trend in risk (according to logistic regression model).

**Table 3:** Selected personal characteristics and prevalence of low back pain (LBP) in medical students.

	With LBP (*n* = 104)	Without LBP (*n* = 395)		
Variable	Number (%)		OR (95% CI)	*P*
Cigarette smoking (ever)	34 (32.7)	68 (17.2)	2.3 [1.4–3.8]	0.001
Body mass index (kg/m2)				
<18.5	10 (9.6)	47 (11.9)	1.0*	
18.5–24.9	85 (81.7)	281 (71.1)	1.4 [0.7–2.9]	0.341
25.0–29.9	9 (8.7)	67 (17.0)	0.6 [0.2–1.7]	0.355
≥30.0	0 (0.0)	0 (0.0)	–	–
*P* trend				0.075
Motor vehicle accident	5 (4.8)	26 (6.6)	0.7 [0.3–1.9]	0.507
Injuries at faculty	8 (7.7)	27 (6.7)	1.1 [0.5–2.6]	0.761
Prolonged standing at faculty	58 (55.8)	204 (51.6)	1.2 [0.8–1.8]	0.454
Prolonged sitting at faculty	98 (94.2)	336 (85.1)	2.9 [1.2–6.8]	0.018
Incorrect body posture	97 (93.3)	325 (82.3)	3.0 [1.3–6.7]	0.008
Lifting/moving heavy loads	32 (30.8)	116 (29.4)	1.1 [0.7–1.7]	0.781
Stress during classes	78 (75.0)	223 (56.5)	2.3 [1.4–3.8]	0.001
Conflicts at faculty	17 (16.3)	51 (12.9)	1.3 [0.7–2.4]	0.365
Recreational sports	26 (25.0)	125 (31.6)	0.7 [0.4–1.2]	0.191
Sport injury	20 (19.2)	85 (21.5)	0.9 [0.5–1.5]	0.611
Incorrect sleeping position	61 (58.7)	145 (36.7)	2.4 [1.6–3.8]	0.008
Housework (washing, etc.)	26 (25.0)	103 (26.1)	0.9 [0.6–1.6]	0.824
Other domestic tasks	14 (13.5)	53 (13.4)	1.0 [0.5–1.9]	0.991
Weather (e.g. cold, humidity, …)	45 (43.3)	145 (36.7)	1.3 [0.8–2.0]	0.221
Draft	63 (60.6)	199 (50.4)	1.5 [0.9–2.4]	0.065
Family history of LBP	74 (71.2)	222 (56.2)	1.9 [1.2–3.1]	0.006
Personal medical history**	39 (37.5)	132 (33.4)	1.2 [0.8–1.9]	0.435
Comorbidity***	11 (10.6)	38 (9.6)	1.1 [0.5–2.3]	0.771

**Notes:**

* Reference category.

** Personal medical history positive for either one or for multiple diseases (depression, anxiety, chronic rheumatic diseases, hypertension, diabetes mellitus, thyroid disease, surgical interventions, etc.).

*** Comorbidity, the presence of more than one disease in a personal medical history.

Abbreviations: OR, Odds Ratio; CI, Confidence Interval; P, Probability value (according to univariate logistic regression analysis); P trend, trend in risk (according to logistic regression model).

Cigarette smoking (*p* = 0.001), prolonged sitting at faculty (*p* = 0.018), incorrect body posture (*p* = 0.008), stress during classes (*p* = 0.001), incorrect sleeping position (*p* = 0.008), and positive family history of LBP (*p* = 0.006) were the variables associated with prevalence of LBP at the time of the study (according to the univariate logistic regression analysis) (Table 3). Also, according to the univariate logistic regression analysis, obesity and draft were associated with prevalence of LBP at *p* < 0.1.

In the multivariate logistic regression model (Table 4), only cigarette smoking (OR = 2.5, 95% CI [1.5–4.2], *p* = 0.001), stress during classes (OR = 1.8, 95% CI [1.1–3.0], *p* = 0.039), incorrect sleeping position (OR = 1.9, 95% CI [1.2–3.1], *p* = 0.006) and family history of LBP (OR = 1.6, 95% CI [1.1–2.7], *p* = 0.047) were independently associated with high prevalence of LBP at the time of the study.

**Table 4:** Factors independently associated with low back pain in medical students: the multivariate logistic regression analysis.

Variable	OR (95% CI)	*P*
Cigarette smoking (ever)	2.5 [1.5–4.2]	0.001
Body mass index (≥25.0)		
<18.5	1.0*	
18.5–24.9	1.5 [0.7–3.2]	0.306
25.0–29.9	0.8 [0.3–2.1]	0.606
≥30.0	–	–
*P* trend		0.172
Prolonged sitting at faculty	1.5 [0.5–4.2]	0.424
Incorrect body posture	1.6 [0.6–4.1]	0.367
Stress during classes	1.8 [1.1–3.0]	0.039
Incorrect sleeping position	1.9 [1.2–3.1]	0.006
Draft	1.1 [0.7–1.8]	0.583
Family history of LBP	1.6 [1.1–2.7]	0.047

**Notes:**

* Reference category.

Abbreviations: OR, Odds Ratio; 95%CI, Confidence Interval; P, Probability value according to multivariate logistic regression analysis; P trend, trend in risk (according to logistic regression model).

## Discussion

Our study showed that one in five medical students at one University in Serbia have LBP. Cigarette smoking, stress during classes, incorrect sleeping position, and LBP in family history were associated with a higher risk for LBP.

The findings of our study showed that LBP is a common occurrence among medical students at a Serbian university. Studies elsewhere have reported similar findings, such as among medical students in Saudi Arabia—23.2% (AlShayhan & Saadeddin, 2018), Malaysia—27.2% (Alshagga et al., 2013). Consistently, point prevalence of LBP among fourth year medical students, was similar in China—17.9% (Smith et al., 2005) and Belgrade University in Serbia—17.2% (Vujcic et al., 2018). A similar prevalence of LBP in our medical students and in the Belgrade study (Vujcic et al., 2018) can be related to a similar academic curricula at all medical faculties in Serbia. LBP was less common among Brazilian medical students with point prevalence of 9.2% (Falavigna et al., 2011). Also, some authors reported point prevalence of 13.0% among medical students at one university in Pakistan (Hafeez et al., 2013). Contrary to these findings, 32.5% of undergraduate students of a medical college in Delhi suffered from LBP at the time of survey (Aggarwal et al., 2013), as well as 34.6% of students studying at health-related faculties at one university in Turkey (Yucel & Torun, 2016). The prevalence of LBP was found to be the highest among medical students in Saudi Arabia: for example, the week prevalence of LBP among medical students ranged from 40.5% at university hospitals in Riyadh (Algarni et al., 2017), to 52.5% among medical students of Jazan University (Dighriri et al., 2019). Variations in the LBP prevalence between studies can be due to differences between populations, study protocols, academic curriculums, as well as cultural, educational, lifestyle or dietary factors.

The significant association between LBP and cigarette smoking in our study was supported by some authors, as they concluded that medical students who smoked were in high risk to develop LBP (Hafeez et al., 2013). However, it was reported in numerous studies that there were no significant differences among medical students in terms of smoking habits and LBP occurrence (Algarni et al., 2017; Aggarwal et al., 2013; AlShayhan & Saadeddin, 2018; Smith et al., 2005; Haroon et al., 2018). On the other hand, cigarette smoking was not examined in a study of LBP in fourth year medical students in Belgrade (Vujcic et al., 2018). One of the most probable explanations for the association between smoking and prevalence of LBP in our study may be the very high prevalence of cigarette smoking among medical students in our study, in contrast to similar studies in the world. In our study, the prevalence of cigarette smoking was high (among students with LBP it was 32.7%, and among students without LBP it was 17.5%), as well as in the study Hafeez et al. (2013) where it was 34.5% among students with LBP, and among students without LBP it was 9.1%. In contrast, in studies where a link between cigarette smoking and LBP was not found (Algarni et al., 2017; Aggarwal et al., 2013; AlShayhan & Saadeddin, 2018; Smith et al., 2005; Haroon et al., 2018) prevalence of smoking was less than 10% in both students with and without LBP. Besides that, the lack of association between cigarette smoking and LBP in the majority of studies can be attributed to young age of medical students in whom LBP has not occurred yet: while in our study and in the study by Hafeez et al. (2013), where an association between cigarette smoking and LBP was found, the average age of participants was 22 and over, in studies where this association was not found, the average age of students was less than 22 (Algarni et al., 2017; Aggarwal et al., 2013; AlShayhan & Saadeddin, 2018; Smith et al., 2005; Haroon et al., 2018; Yucel & Torun, 2016). Also, study participants were medical students in whom education is encouraging them to give up smoking.

Family history of LBP was independently linked to LBP prevalence in medical students in our study. This finding is consistent with findings of some previous reports (Alshagga et al., 2013; Aggarwal et al., 2013; AlShayhan & Saadeddin, 2018), but not all (Haroon et al., 2018). On the other hand, Vujcic et al. (2018) did not collect data about family history of LBP in medical students in Belgrade. Also, some authors revealed that the severity of LBP was higher in health university students with a history of hereditary spinal diseases (Yucel & Torun, 2016). Therefore, particularly those medical students with positive family history of LBP or other musculoskeletal disorders constitute a vulnerable population in which LBP prevention measures should be implemented still during studies.

The significant association between academic stress and LBP in this study is confirmed by the results of some studies (Haroon et al., 2018; Vujcic et al., 2018; Smith et al., 2005), but results were inconsistent (Aggarwal et al., 2013). It is reported that feeling discomfort on bed is strongly associated with LBP (AlShayhan & Saadeddin, 2018). In this study, the results show a significantly higher point prevalence of LBP among medical students who have incorrect sleeping position. This result can be attributed to the fact that uncomfortable sleeping increases compression load on the spine.

Some other studies have indicated an association between academic year and LBP among medical students (Algarni et al., 2017; Alshagga et al., 2013; Falavigna et al., 2011), but such findings were not reported by our and other studies (Dighriri et al., 2019). While our study did not find a significant impact of physical activity (such as recreational sports, housework, etc.), numerous studies have consistently been showing that regular exercise provides significantly good effects on LBP occurrence, but without statistical significance (Algarni et al., 2017; Dighriri et al., 2019; AlShayhan & Saadeddin, 2018; Falavigna et al., 2011; Yucel & Torun, 2016; Smith et al., 2005; Haroon et al., 2018). On the other hand, the association between certain factors (female gender, history of trauma, etc.) and risk of LBP among medical students which has been very consistent in some studies (Algarni et al., 2017; Dighriri et al., 2019; Haroon et al., 2018; Smith & Leggat, 2007; Moroder et al., 2011) was not confirmed in our study.

Certain disagreements between the results of our study and other studies of LBP prevalence and associated factors in medical students can be explained by differences in the observed populations (by gender, age, study year, lifestyle habits, behavior factors, family history, comorbidity), study design, as well as differences in definition and measures of LBP. Also, in some studies prevalence over different time periods over which the information was gathered (currently, last week, etc.) was described which could have led to recall bias due to misclassification and could have made comparing results difficult. Also, the measurement of the exposure factors may have introduced bias that potentially explains the differences noted between this and previous research. Nevertheless, this study has helped identify some key variables (primarily, three modifiable factors: cigarette smoking, stress during classes, incorrect sleeping position) which are important for finding the best preventive strategies for LBP in medical students. Furthermore, longitudinal studies of LBP prevalence in medical students before enrollment at the university, during studies and after graduation and characteristics of medical students should be performed in order to evaluate the link between LBP and associated factors.

### Strengths and limitations of the study

To the best of our knowledge, our study is one of the first studies on LBP in medical students in Serbia. Even though the response rate was satisfactory, this study has several possible limitations. Data in this study were based on anonymous self-reports, but information bias cannot be ruled out. Limitation of the present study includes sampling bias, because only students who attended lectures were involved. Further, there remains the possibility of participant over- or underreporting their LBP. Even though surveys using self-report data can be inaccurate, medical students were in a position to understand both the definitions we used in the study and the importance of giving accurate and true responses to health survey due to their acquired medical knowledge. Also, since the study was conducted at one faculty, the results may not be representative of all medical students in Serbia. Finally, one important limitation of our study is the cross-sectional design, with all known shortcomings of such studies in identifying factors predicting LBP.

## Conclusions

Our study showed that LBP is a frequent condition in medical students at one University in Serbia. Although our study identified that cigarette smoking, stress during classes, incorrect sleeping position and LBP in family history were associated with high prevalence of LBP, these associations should be confirmed by epidemiological longitudinal studies. A better understanding of etiology of LBP can help in establishing preventive measures for LBP in medical students as future health professionals.

## Supplemental Information

10.7717/peerj.11055/supp-1 Supplemental Information 1 Questionnaire LBP—English.

10.7717/peerj.11055/supp-2 Supplemental Information 2 Questionnaire LBP—Serbian.

## Additional Information and Declarations

### Data Availability

The following information was supplied regarding data availability: The data is available at Harvard Dataverse: Ilic, Milena, 2020, “PREVALENCE AND RISK FACTORS FOR LOW BACK PAIN IN STUDENTS OF MEDICINE AND PHARMACY—A PREVALENCE STUDY”, DOI 10.7910/DVN/PEIPHC, Harvard Dataverse, V1, UNF:6:vXc61cTlo8AgXXPci+HzoA== [fileUNF]
