# Supplementary material for: Prevalence and correlates of low back pain among undergraduate medical students in Serbia, a cross-sectional study
Source: PeerJ. 2021 Mar 8;9:e11055. doi: 10.7717/peerj.11055 (PMC7950191; doi:10.7717/peerj.11055)
Supplement: Supplemental Information 1 [file peerj-09-11055-s001.doc]

**FACULTY OF MEDICAL SCIENCES KRAGUJEVAC**

**PREVALENCE AND RISK FACTORS FOR LOW BACK PAIN IN STUDENTS OF MEDICINE AND PHARMACY – A PREVALENCE STUDY**

1. **DEMOGRAPHIC CHARACTERISTICS AND HABITS**

1. Year of birth: ________________

2. Gender: **Male Female**

3. Completed secondary school: **a) grammar school b) secondary medical school c) other**

4. Integrated academic studies of medicine: __________________________________________________

5. Study year

6. Subjects

7. BMI

8. Do you smoke? **YES NO**

1. **FACTORS ASSOCIATED WITH PREVALENCE OF LOW BACK PAIN**

**This table lists various factors which could be the reason for low back pain. Have you ever been exposed to any of the listed factors or have you ever been in the listed circumstances?**

| **FACTORS** | **YES** | **NO** | **POSSIBLY** |
| --- | --- | --- | --- |
| 09. Weather (e.g. cold, humidity, …) |  |  |  |
| 10. Air condition/draft |  |  |  |
| 11. I have family members with low back pain |  |  |  |
| 12. Motor vehicle accident |  |  |  |
| 13. Sports injuries |  |  |  |
| 14. Injuries at faculty |  |  |  |
| 15. Prolonged standing at faculty |  |  |  |
| 16. Prolonged sitting at faculty |  |  |  |
| 17. Incorrect body posture at faculty |  |  |  |
| 18. Lifting/moving heavy loads at faculty |  |  |  |
| 19. Stress during classes |  |  |  |
| 20. Conflicts at faculty |  |  |  |
| 21. Incorrect sleeping position |  |  |  |
| 22. Recreational sports |  |  |  |
| 23. Domestic tasks: window washing |  |  |  |
| 24. Domestic tasks: ironing |  |  |  |
| 25. Domestic tasks: state any other ___________________ |  |  |  |
| 26. Surgical interventions |  |  |  |
| 27. Any other reason state here____________ |  |  |  |

**28. Personal medical history for chronic diseases (**depression, anxiety, some other psychiatric illness, rheumatic disease, arterial hypertension, ischemic heart disease, diabetes mellitus, thyroid disease, asthma, anemia, migraine, sinusitis, myopia**):**

**NO YES**

**III. PERSONAL HISTORY FOR LOW BACK PAIN**

29. Do you now have low back pain which lasts for at least 1 day (with or without pain referred into one or both lower limbs)?  **NO YES**
